# An Evaluation of Culture Results during Treatment for Tuberculosis as Surrogate Endpoints for Treatment Failure and Relapse

**DOI:** 10.1371/journal.pone.0063840

**Published:** 2013-05-08

**Authors:** Patrick P. J. Phillips, Katherine Fielding, Andrew J. Nunn

**Affiliations:** 1 Medical Research Council Clinical Trials Unit, London, United Kingdom; 2 London School of Hygiene and Tropical Medicine, London, United Kingdom; San Francisco General Hospital, University of California San Francisco, United States of America

## Abstract

It is widely acknowledged that new regimens are urgently needed for the treatment of tuberculosis. The primary endpoint in the Phase III trials is a composite outcome of failure at the end of treatment or relapse after stopping treatment. Such trials are usually both long and expensive. Valid surrogate endpoints measured during or at the end of treatment could dramatically reduce both the time and cost of assessing the effectiveness of new regimens.

The objective of this study was to evaluate sputum culture results on solid media during treatment as surrogate endpoints for poor outcome. Data were obtained from twelve randomised controlled trials conducted by the British Medical Research Council in the 1970s and 80s in East Africa and East Asia, consisting of 6974 participants and 49 different treatment regimens.

The month two culture result was shown to be a poor surrogate in East Africa but a good surrogate in Hong Kong. In contrast, the month three culture was a good surrogate in trials conducted in East Africa but not in Hong Kong. As well as differences in location, ethnicity and probable strain of *Mycobacteria tuberculosis*, Hong Kong trials more often evaluated regimens with rifampicin throughout and intermittent regimens, and patients in East African trials more often presented with extensive cavitation and were slower to convert to culture negative during treatment.

An endpoint that is a summary measure of the longitudinal profile of culture results over time or that is able to detect the presence of *M. tuberculosis* later in treatment is more likely to be a better endpoint for a phase II trial than a culture result at a single time point and may prove to be an acceptable surrogate. More data are needed before any endpoint can be used as a surrogate in a confirmatory phase III trial.

## Introduction

Tuberculosis (TB) is one of the world's oldest infectious disease and over the centuries has been responsible for more mortality, morbidity and human suffering that any other[1]. Though an effective cure is available today for no more than US $20, it is estimated that there are over 9 million new cases and almost 2 million people die every year from TB[2]. The six month standard regimen for drug-susceptible TB has been shown to be highly efficacious in clinical trials[3], but such results are rarely achieved in practice[4]. New treatment regimens are urgently needed to reduce the duration of treatment for drug-susceptible TB and effectively treat multi-drug resistant TB (MDR-TB).

TB is almost unique among bacterial infections in that failure to culture the bacilli is not necessarily indicative of cure. An effective regimen is one which renders patients culture negative by the end of treatment, but also prev_ENREF_5ents subsequent relapse. Clinical trials to evaluate new regimens for the treatment of TB therefore commonly involve follow-up beyond the end of treatment of 18–24 months[5]. A surrogate endpoint measured during or at the end of treatment could be used as a substitute for the currently used composite clinical endpoint of treatment failure and relapse in a phase III clinical trial[5] thereby substantially shortening the trial duration and speeding overall drug development.

A biomarker is any marker ‘objectively measured and evaluated as an indicator of normal biological processes, pathogenic processes, or pharmacologic responses to a therapeutic intervention’[6]. A surrogate endpoint is a biomarker that *fully captures* the effect of the trial intervention on the clinical endpoint and ‘for which a test of the null hypothesis of no relationship to the treatment groups under comparison is also a valid test of the corresponding null hypothesis based on the true [clinical] endpoint’[7]. Such an endpoint can only be defined in the context of a comparison of different treatment arms in a randomised controlled trial. _ENREF_7One of the most important principles for evaluating a putative surrogate is that mere correlation does not imply surrogacy[8], [9].

There have been several reviews on surrogate endpoints and biomarkers of TB treatment response[10]–[12]_ENREF_8. Perrin et al.[10] summarised the potential markers currently available that could describe a patient's response to treatment (*biomarkers of treatment response*) but no formal evaluation of surrogate endpoints was provided. The two month culture was shown to be a risk factor for relapse in two trials[13], [14] but there was, however, no attempt to formally evaluate the endpoint as a surrogate endpoint.

Perrin et al. also referred to a review by Mitchison[15] expanding on earlier correspondence[16] which addressed the relationship between relapse rates and rates of culture positivity across several clinical trials. It is not clear which statistical methods were used; the results indicate a relationship suggestive of surrogacy, but with a note of caution that ‘the most effective time for measuring sputum conversion may vary according to the drug under test.’[15] The report from an expert consultation meeting on biomarkers in TB organised by WHO Tropical Disease Research (TDR) also identified the two month culture result as being currently accepted as a surrogate for treatment outcome, but again without any formal evaluation to support this[17]. A recent systematic review of sputum monitoring during TB treatment for predicting outcome found the two month culture had modest specificity but low sensitivity for predicting failure and relapse [18].

The objective of the present study was to evaluate sputum culture results during treatment as potential surrogate endpoints for long term outcome in the treatment of pulmonary TB using appropriate statistical methodology. Treatment comparisons are required for evaluating a surrogate endpoint and therefore it is necessary to use data from randomised controlled clinical trials where culture results during treatment are available as well as follow up for relapse for a minimum of 18 months. To achieve this objective, data were used from selected TB clinical trials conducted by the British Medical Research Council (BMRC) during the 1970s and 1980s.

## Methods

### Ethics Statement

This study of past clinical trial data was approved by the ethics committee of the London School of Hygiene and Tropical Medicine.

### Selection of studies

Individual patient data were available from all TB clinical trials that were conducted by the BMRC in East Africa and East Asia[3]. These trials provided much of the evidence for the short course regimens which are standard treatment today[3], [19], [20]. The advantage of using data only from BMRC trials is that the clinical and bacteriological protocols were largely unchanged throughout the programme of trials and the level of homogeneity was therefore high. These data are therefore ideal for the evaluation of culture results during treatment as surrogate endpoints.

These large multi-centre randomised controlled trials included high quality laboratory data with frequent follow-up sampling after the end of treatment on smear positive patients with pulmonary TB. All trials included regimens comprised of various combinations of first-line drugs available and recommended for use today[2]: isoniazid, rifampicin, ethambutol and pyrazinamide, in addition to two drugs no longer used in first-line regimens: thiacetazone and streptomycin. From this pool of trials, treatment arms of duration other than six months (the duration of the WHO-recommended regimen in use today) have been excluded as were trials of regimens all with less than 2% relapses as the small numbers of relapses yield too little information for evaluating surrogates. The full list of treatment arms included is given in Table 1.

**Table 1:** List of trials and treatment arms included in this study.

Trial		Year of Start	Treatment arms included (first regimen nominated as control)
East Africa	1	1970	6SH, 6SHT, 6SHZ, 6SHR
	2	1972	6HR, 2SHRZ/4TH, 2SHRZ/4SHZ_2_, 6SHR
	3	1974	2SHR/4TH, 1SHRZ/5TH, 2SHRZ/4TH, 1SHRZ/5SHZ_2_
	4†	1976	2SHRZ/4H, 2HRZ/4H, 2SHRZ/4HR, 2SHRZ/4HZ, 2SHRZ/4HRZ
	5	1978	2SHRZ/4H, 2SHRZ/4HZ, 2SHRZ/4HR
	6	1978	2SHRZ/4H, 2SHRZ/4TH
Hong Kong	1	1972	6SHZ_2_, 6SHZ, 6SHZ_3_
	2	1974	2SHRE/4SHE_2_, 2SHRZ/4SHZ_2_, 4SHRZ_3_/2SHZ_2_
	3	1977	6HRSE_3_, 6HRZE_3_, 6HRSZ_3_, 6HRSZE_3_, 6HRZE
	4*	1979	2HREZ_3_/4HRE_3_, 6SHRE_3_(2wZ_1_), 6SHRE_3_, 6SHRE_3_(4wZ_1_), 6SHRE_3_(8wZ_3_), 6SHRE_3_(2wZ_3_), 6SHRE_3_(8wZ_1_), 6SHRE_3_(4wZ_3_)
Singapore	1	1973	2SHRZ/4HR, 2SHRZ/4HRZ
	3	1983	2S(HRZ)_C_/4HR_3_, 2(HRZ)_C_/4HR_3_, 1S(HRZ)_C_/5HR_3_, 1SHRZ/5HR_3_, 2HRZ/4HR_3_, 2SHRZ/4HR_3_

Trial numbering corresponds to numbering in a comprehensive review of all MRC studies[3], where full references for trial report(s) are listed. Trials conducted in East and Central Africa are listed in Table 1.7, trials conducted in Hong Kong in Table 1.8 and trials conducted in Singapore in Table 1.9 of the review[3].

† This trial was actually of 4 month regimens, but was terminated earlier than planned and patients still on treatment at that time were continued to 6 months of treatment. The results of those on 6 months of treatment were presented in a later publication[44].

* The results of this trial were never published (personal communication, DA Mitchison) and it is therefore not included in the tables of MRC studies in Fox, Ellard and Mitchison[3]. For treatment notation: S  =  Streptomycin, H  =  Isoniazid, T  =  Thiacetazone, Z  =  Pyrazinamide, R  =  Rifampicin, E  =  Ethambutol. Where the regimen has distinct intensive and continuation phases, these are separated by a forward slash with the leading number corresponding to the duration in months. The subscript indicates the number of doses given weekly; the absence of subscript indicates daily dosing. For example, 2SHRE/4SHE_2_ consists of a 2 month intensive phase of 4 drugs given daily followed by a 4 month continuation phase of 3 drugs each given twice-weekly. The subscript *C* indicates the drugs were given in a combined formulation. In the fourth Hong Kong study pyrazinamide given once or thrice weekly was added to some of the regimens for the first 2, 4 or 8 weeks. This is indicated by the text in parentheses where, for example, 4wZ_1_ indicates that once-weekly pyrazinamide was added only for the first 4 weeks.

### Clinical and Surrogate Endpoint Definitions

A treatment failure was defined as heavy growth on culture (at least 20 colonies) at month 5 or 6 and a relapse defined as two cultures with heavy growth within three consecutive months or three positive cultures with any growth (one colony or more) within four consecutive months, following the end of treatment. The clinical endpoint was a combined endpoint of treatment failure at the end of treatment or relapse in follow-up hereafter referred to as poor outcome. In the original publications, these were usually presented as two separate endpoints, but were combined in this study in a composite endpoint to reflect the endpoint currently used in phase III TB trials[5], [21], [22]. The bacteriological definitions of treatment failure and relapse were taken from with the original trial reports[3], with the exception that ‘heavy growth’ was sometimes defined as at least 5, 10 or 20 colonies. Heavy growth of at least 20 colonies was chosen for consistency across trials. Default or death from a non-TB cause during treatment were classified as a missing clinical endpoint. If a patient was lost to follow-up after a single positive culture, they were classified as a relapse if no further data were available.

Cultures on solid media during treatment were available monthly and were recorded on a semi-categorical scale: negative, 0–19 colonies, 20–100 colonies, more than 100 colonies, or confluent growth. Three endpoints were evaluated as potential surrogates: i) a positive culture of at least 20 colonies at month one, ii) a positive culture of any growth at month two and iii) a positive culture of any growth at month three. Most patients were still culture positive after one month of treatment, so the endpoint selected for this time point is a positive culture of at least 20 colonies. Insufficient patients were culture positive at month four for that to be a useful endpoint.

### Statistical Methods

Patients identified as having additional extra-pulmonary TB were often withdrawn from the trials and so data on treatment outcomes were often missing. For this reason, this small number of patients were excluded from the analysis. Patients with negative cultures at baseline were also excluded.

Culture results during treatment were evaluated as surrogate endpoints for poor outcome using a two stage approach based on a frequentist application of the Bayesian methods developed to evaluate CD4 count as a potential surrogate the development of AIDS or death[23]. Both stages are repeated for each of the three candidate surrogates. The first stage involves analysis at the trial participant-level estimating the treatment effect on the surrogate endpoint α_ij_ (expressed as the log odds ratio of a positive culture) and the treatment effect on the clinical endpoint β_ij_ (expressed as the log odds ratio of a poor outcome) for each treatment comparison j, of an experimental arm with the control arm in each trial i. Many of the trials did not have a pre-specified ‘control regimen’ and so, unless the control arm was obvious, the arm with the highest proportion of poor outcomes was identified as the nominal control. This was done so that the difference in risk of poor outcome between the experimental and control regimens is greatest and therefore the treatment ordering is such that the most amount of information is available for evaluating culture results as surrogate endpoints. Where two or more arms in a trial had the same treatment for the first one or two months, the control arm was selected to limit the number of comparisons of such arms.

The second stage involves analysis at the treatment-comparison-level fitting a linear regression model with β_ij_ as the response and α_ij_ as the explanatory variable. Since the pairs (α_ij_, β_ij_) are estimated with varying precision, the model is fitted with weights equal to the inverse of the mean of the variances of the α_ij_ and β_ij_ for each i and j. Robust standard errors are used to account for the clustering of treatment comparisons within trials and the intercept term in the linear model is constrained to be zero since each treatment comparison corresponds to comparisons of different treatment regimens and therefore a non-zero intercept has no meaning. The treatment effect on poor outcome is also plotted against the treatment effect on the surrogate with the diameter of the circles corresponding to the precision of the estimates. Estimates with greater precision, and therefore larger weight in the linear model, are represented by larger circles. The proportion of variation in β_ij_ explained by α_ij_ in this situation is called the trial-level proportion of variation explained, R^2^_trial_. This is an established metric for evaluating surrogate endpoints[24], and based on a number of examples of the use of this metric, an R^2^_trial_≥0.80 could be considered as evidence for a surrogate being ‘good’ and R^2^_trial_≥0.95 being ‘very good’[24]. The analyses were repeated incorporating adjustment for important baseline patient risk factors (including smear and culture status, pre-treatment drug resistance, extent of cavitation, weight, age and sex) in the first stage of the two-stage analysis.

## Results

### Baseline Characteristics

Table 2 summarises the baseline characteristics of the trials and patients included in this study. Data were included from 12 trials, yielding 49 trial arms and 37 total possible treatment comparisons. Relapse rates and details of the treatment arms can be found elsewhere[3] along with references of the individual trial reports. Patients in the Hong Kong trials were more likely to have pre-treatment resistance to isoniazid (9% overall) or streptomycin (10%) than in the East African trials, 7% isoniazid resistance and 3% streptomycin resistance. Patients in the East African trials were much more likely to present with extensive or gross cavitation (44% overall) than in the Hong Kong trials (5% overall).

**Table 2:** Summary of baseline characteristics of trial participants included in the analysis.

							Drug Resistance		Bacteriology, classified as heavy (20 colonies or more on culture, 3+grading on smear) N (%)		Radiography, classified as ‘extensive’ or ‘gross’ using a standardised grading[45], [46] N (%)	
Trials		Participants	Treatment arms	Male N (%)	Age Median (IQR)	Weight/kg Median (IQR)	Isoniazid N (%)	Streptomycin N (%)	Culture	Smear	Extent of cavitation	Extent of disease
East Africa	1	761	4	520 (68%)	30 (24–41)	49 (43–54)	65 (9%)	16 (2%)	733 (98%)	279 (37%)	377 (59%)	326 (50%)
	2	902	4	566 (63%)	32 (25–44)	48 (44–53)	55 (6%)	36 (4%)	854 (96%)	217 (24%)	468 (54%)	454 (52%)
	3	421	4	268 (64%)	30 (25–40)	47 (42–52)	28 (7%)	17 (4%)	406 (96%)	157 (37%)	242 (59%)	200 (49%)
	4	310	5	197 (64%)	34 (25–44)	48 (43–51)	25 (8%)	4 (1%)	299 (97%)	96 (31%)	48 (17%)	99 (35%)
	5	533	3	339 (64%)	30 (23–40)	47 (42–52)	28 (5%)	9 (2%)	509 (97%)	241 (46%)	56 (12%)	220 (45%)
	6	296	2	208 (71%)	34 (25–45)	49 (43–54)	24 (8%)	11 (4%)	264 (91%)	47 (16%)	N/A	N/A
Hong Kong	1	246	3	175 (71%)	37 (23–50)	46 (41–51)	27 (11%)	33 (13%)	220 (89%)	129 (52%)	28 (11%)	51 (21%)
	2	369	3	288 (78%)	36 (21–55)	47 (43–52)	46 (12%)	48 (13%)	343 (93%)	125 (34%)	48 (13%)	67 (18%)
	3	1142	5	824 (72%)	31 (22–52)	N/A	89 (8%)	104 (9%)	1097 (96%)	298 (26%)	18 (2%)	126 (11%)
	4*	1489	8	1061 (71%)	32 (23–50)	48 (44–53)	134 (9%)	147 (10%)	1428 (97%)	503 (34%)	N/A	N/A
Singapore	1	198	2	129 (65%)	43 (29–54)	45 (41–50)	4 (2%)	9 (5%)	192 (97%)	123 (62%)	4 (2%)	48 (24%)
	3	307	6	199 (65%)	38 (25–51)	49 (42–55)	6 (2%)	9 (3%)	302 (98%)	161 (52%)	N/A	N/A
**Total**		**6974**	**49**	**4774 (69%)**	**33 (23–47)**	**48 (43–53)**	**531 (8%)**	**443 (6%)**	**6647 (96%)**	**2376 (34%)**	**1289 (28%)**	**1591 (34%)**

Some data elements were not available (N/A) in certain trials. Trial numbering corresponds to numbering in a comprehensive review of all MRC studies[3], where full references for trial report(s) are listed. Trials conducted in East and Central Africa are listed in Table 1.7, trials conducted in Hong Kong in Table 1.8 and trials conducted in Singapore in Table 1.9 of the review[3].

* The results of this trial were never published (personal communication, DA Mitchison) and it is therefore not included in the tables of MRC studies in Fox, Ellard and Mitchison[3]. IQR - Inter-Quartile Range.

### Evaluating the candidate surrogates

Treatment for TB is usually described in two phases, the *intensive phase* where three to four drugs are given together followed by the *continuation phase* where, typically, only two of these drugs are continued until the end of treatment. Some treatment comparisons involved regimens with the same drug combination in the first few months of treatment. These comparisons were therefore excluded from the evaluation of the one and two month cultures. In summary, of the 37 total possible treatment comparisons from 49 trial arms, 32 treatment comparisons were used to evaluate a positive culture of heavy growth at month one, 33 to evaluate the month two culture result and 35 the month three culture result, as surrogate endpoints. All treatment comparisons involved substantial changes to the regimen in the first few months.

**Figure 1:**
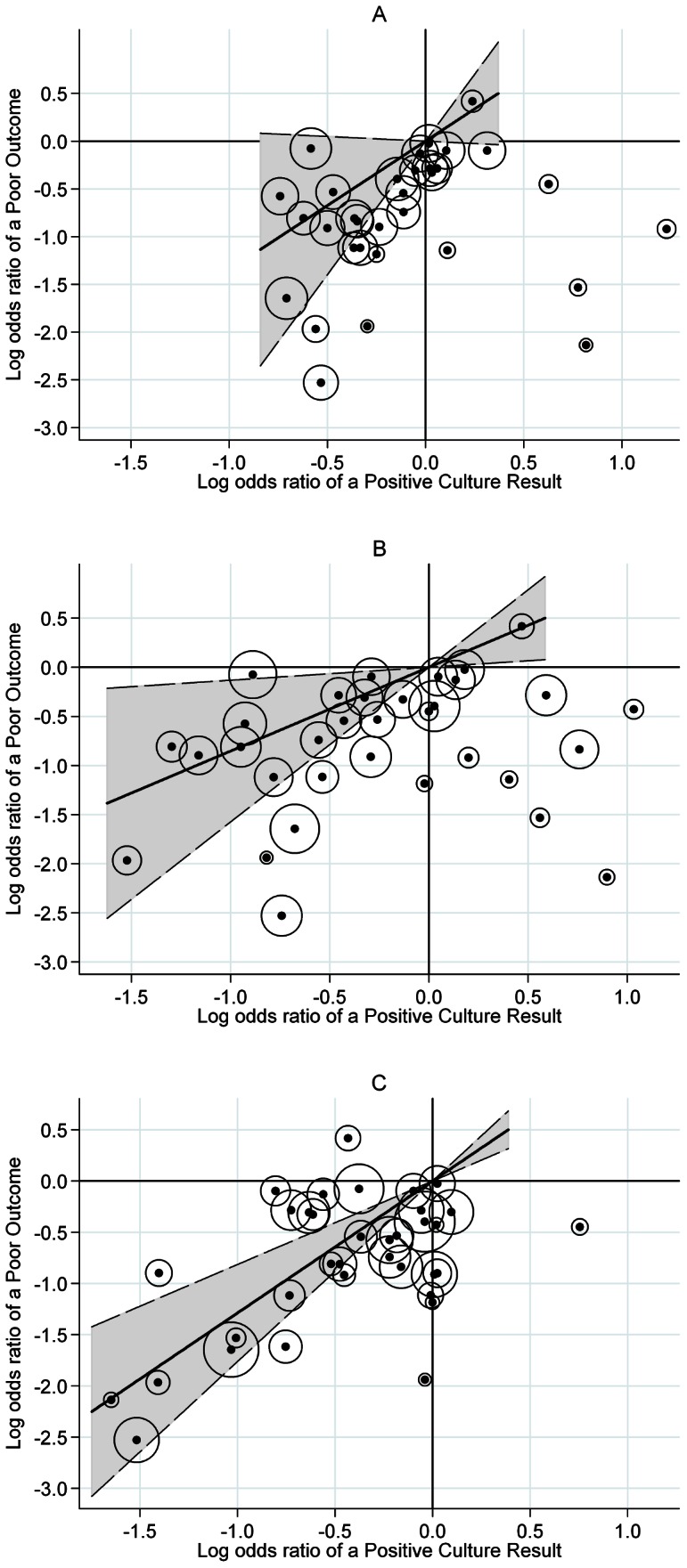
Analysis of culture results as surrogate endpoints across all trials. **A**. Month 1 (a positive culture with heavy growth, at least 20 colonies), R^2^_trial_ = 0.36. **B**. Month 2 (a positive culture with any growth), R^2^_trial_ = 0.36. **C**. Month 3 (a positive culture with any growth), R^2^_trial_ = 0.69. Logs odds ratio of a poor outcome plotted against log odds ratio of a positive culture. Fitted line is weighted by the precision of the estimates, and this precision is represented by the diameter of the circles around each point. The dotted line represents the 95% confidence interval on the slope.

Figure 1 and the first three rows of Table 3 show the results of the second stage of the analysis for all trials overall for the three potential surrogate endpoints.

**Table 3:** Estimates of the slope of the fitted line and of the proportion of explained variation from the model from the second stage.

Analysis	Month of Culture^a^	Trials	Treatment Comparisons^b^	Slope (95% CI)	R^2^_trial_
Overall	1	9	32	1.35 (−0.10, 2.80)	0.36
	2	9	33	0.85 (0.13, 1.57)	0.36
	3	11	35	1.29 (0.82, 1.76)	0.69
East Africa trials only	1	4	13	1.13 (−1.82,4.11)	0.29
	2	4	13	0.76 (−1.57,3.09)	0.19
	**3**	**6**	**16**	1.61 (1.38,1.83)	**0.81**
Hong Kong trials only	1	4	15	1.98 (−0.92,4.05)	0.68
	**2**	**4**	**15**	0.99 (0.82,1.16)	**0.86**
	3	4	15	0.82 (0.09,1.56)	0.62
Singapore trials only	1	1	4	0.19 (−4.95,5.33)	<0.01
	2	1	5	−0.06 (−2.31,2.20)	<0.01
	3	1	4	−0.52 (−5.09,4.05)	0.04

CI - Confidence Interval; R^2^_trial_ - trial-level proportion of variation.

a The month 1 endpoint evaluated was a positive culture with heavy growth (at least 20 colonies). The month 2 and 3 endpoints evaluated were a positive culture with any growth (at least 1 colony).

b Due to similarities in regimens in the first few months of treatment, not all 37 treatment comparisons could be used for each analysis.

There is considerable scatter about the fitted line in Figures 1A and 1B with the proportions of variation explained, R^2^_trial_, only 0.36 in each case. This can be interpreted as a weak relationship between the treatment comparison on the clinical endpoint of a poor outcome and the treatment comparison on the candidate surrogate endpoint meaning that it would not be possible to use the effect of a treatment regimen on the candidate surrogate to accurately predict the effect of the treatment on the proportion of poor outcomes.

Apart from the groupings around the origin in figure 1B (showing no difference between treatment on either endpoint), there are at least seven points in the lower right quadrants indicating that the treatment direction on a poor outcome is opposite to that on the candidate surrogate. The corresponding treatments would appear to be inferior to the control when evaluating the candidate surrogate, but superior to the control when evaluating the proportion of poor outcomes.

There is less scatter about the fitted line in Figure 1C than in Figures 1A and 1B and this is reflected in a proportion of explained variation considerably higher at 0.69 and the narrowest 95% confidence interval on the slope. Excluding the clustering around the origin, there is only one point in the lower right quadrant and one in the upper left.

### Patient-level adjustment for baseline factors

The analyses were repeated incorporating adjustment in the first stage of the analysis for important baseline patient risk factors (including smear and culture status, pre-treatment drug resistance, extent of cavitation, weight, age and sex) with no substantial differences in the result (data not shown).

### Analysis by geographical region

**Figure 2:**
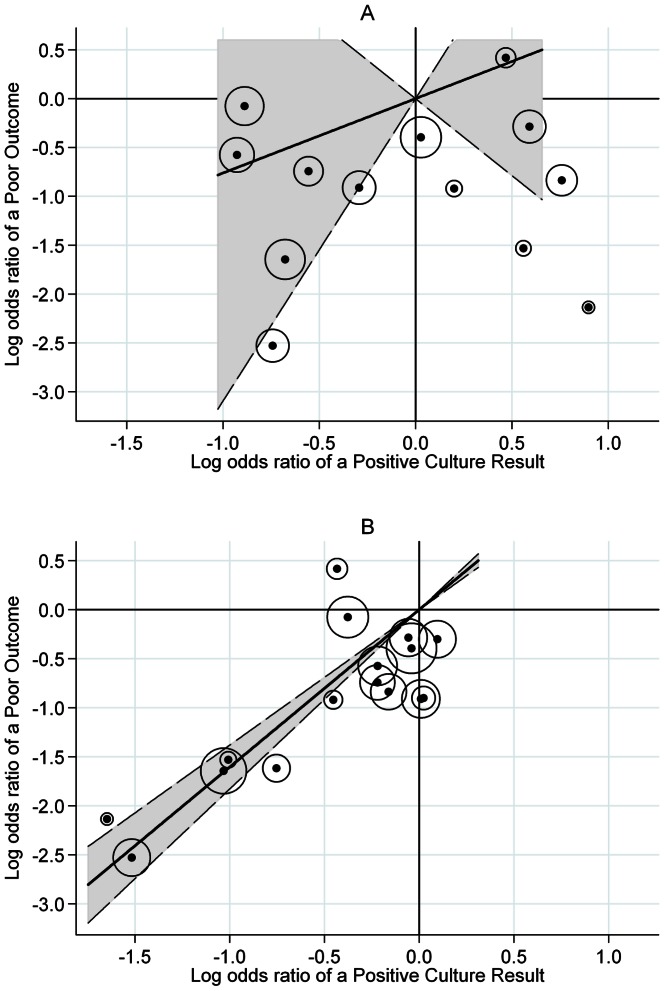
Sub-group analysis by geographical region: East African trials. **A.** Month 2 restricted to East African trials, R^2^_trial_ = 0.19. **B**. Month 3 restricted to East African trials, R^2^_trial_ = 0.81. Logs odds ratio of a poor outcome plotted against log odds ratio of a positive culture. Fitted line is weighted by the precision of the estimates, and this precision is represented by the diameter of the circles around each point. The dotted line represents the 95% confidence interval on the slope.

**Figure 3:**
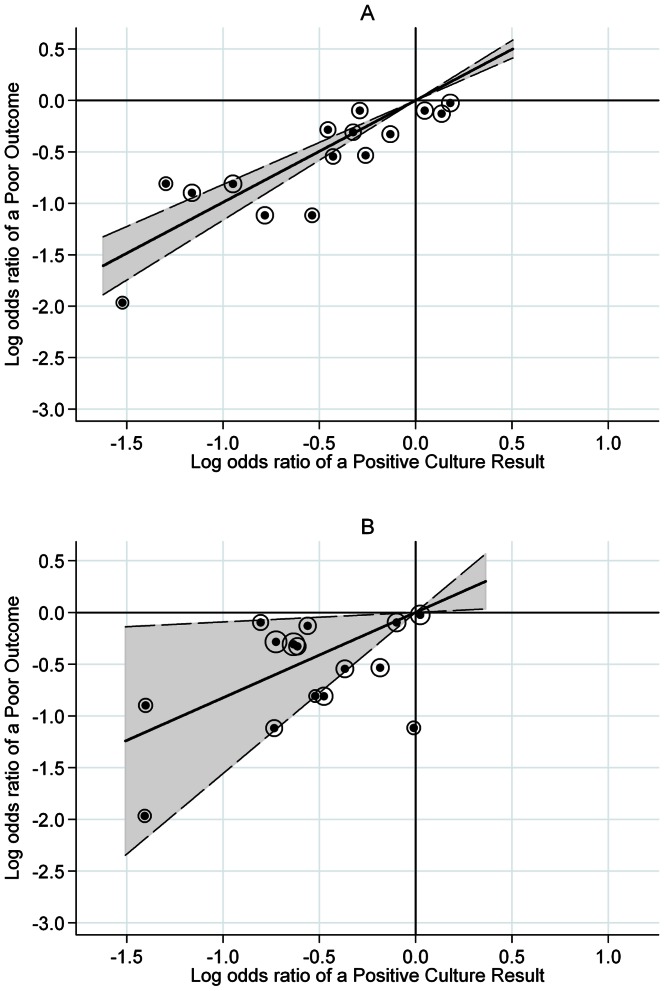
Sub-group analysis by geographical region: Hong Kong trials. **A.** Month 2 restricted to Hong Kong trials, R^2^_trial_ = 0.86. **B**. Month 3 restricted to Hong Kong trials, R^2^_trial_ = 0.62. Logs odds ratio of a poor outcome plotted against log odds ratio of a positive culture. Fitted line is weighted by the precision of the estimates, and this precision is represented by the diameter of the circles around each point. The dotted line represents the 95% confidence interval on the slope.

Most of the trials were conducted across two separate geographical regions: East Africa and Hong Kong. There is evidence of different relapses rates between clinical trials conducted in these two regions using the same treatment regimen on three separate occasions[3]. Therefore, the three candidate surrogate endpoints were evaluated in trials separately for Hong Kong and East Africa. The results are shown in Figures 2 and 3 and Table 3. Too few treatment comparisons (all from one trial) were available from trials in Singapore to draw any clear conclusions.

At months one and two, analysis restricted to data from East Africa shows great variation about the line, with the proportion of variation explained 0.29 and 0.19, respectively, with very wide confidence intervals. This contrasts with analysis of the month three culture in East Africa, where there is a clear linear trend (Figure 2B, proportion of variation explained 0.81).

Six points lie in the lower right quadrant in Figure 2A, indicating an effect of treatment on the two month culture result that is in the opposite direction to the effect on the proportion of poor outcomes. Of these, two correspond to the addition of rifampicin beyond two months, two to the addition of pyrazinamide beyond two months and two to little or no change beyond 2 months.

In contrast to the East African graphs, a linear trend is more apparent for all months in the graphs restricted to data from Hong Kong only. At months one and three the proportions of explained variation are reasonably high at 0.69 and 0.62 respectively though the widths of the confidence intervals are also very wide. The best fit is at month two with a narrow 95% confidence interval around the slope, a high proportion of explained variation at 0.86, and no points outside the lower left quadrant, except for three which are very close to the origin (Figure 3A).

Fitting one model allowing for different slopes for trials from Hong Kong and East Africa, there was no evidence for interaction with p = 0.46 and p = 0.75 for months one and two, respectively. At month three, there was evidence for a difference in slopes between trials from Hong Kong and trials from East Africa, p = 0.015.

Table 4 shows a summary of some of the differences by geographical region. 73% of the treatment comparisons in the Hong Kong trials were of two regimens that included rifampicin throughout compared to only 6% of the treatment comparisons in the East African trials. Similarly, all of the regimens evaluated in the East African trials had daily dosing in the intensive phase of treatment compared to only 7% in the Hong Kong trials and 88% in the continuation phase in East African trials compared to none in the Hong Kong trials.

**Table 4:** Summary of characteristics of treatment comparisons and trial participants by geographical region.

				Daily dosing in both regimens throughout phase N(%)		Participants culture positive N (%)			
Region	Trials	Treatment Comparisons	Six months of rifampicin in both regimens N(%)	Intensive Phase	Continuation Phase	Month 1	Month 2	Month 3	Month 4
East Africa	6	16	1 (6%)	16 (100%)	14 (88%)	1988 (70%)	829 (29%)	292 (10%)	158 (6%)
Hong Kong	4	15	11 (73%)	1 (7%)	0 (0%)	1391 (46%)	445 (15%)	113 (4%)	78 (3%)
Singapore	2	6	6 (100%)	6 (100%)	1 (17%)	246 (51%)	34 (7%)	7 (1%)	5 (1%)

Culture conversion occurred earlier on average in Hong Kong compared to East Africa. 15% of patients at two months and only 4% of patients at three months were still culture positive in Hong Kong compared with 29% and 10% respectively in East Africa.

Repeating the analyses for comparisons of two regimens that contained rifampicin throughout treatment resulted in 0.67 and 0.46 proportion of variation explained for the 2 month and 3 month cultures respectively.

## Discussion

The two month culture has variously been described as ‘the main surrogate marker [for sterilizing activity]’[25], a ‘currently available surrogate marker of relapse rates’[26], ‘probably the best available surrogate marker for the relapse rate’[27] and ‘an index of efficacy of anti-TB regimens’[28]. These conclusions are based on limited published evidence and a varied understanding of what is meant by a surrogate.

Using the definitions of surrogacy outlined in the introduction, this study has shown that the two month culture appeared to be a good surrogate endpoint using data from the Hong Kong trials and the three month culture was suggestive of a good surrogate endpoint using data from the East African trials, but the reverse was not true.

There were no substantial differences in the results on adjustment for patient-level baseline factors in the first stage of the analysis. This was not unexpected as each treatment comparison was a comparison of randomised groups which would be likely to be reasonably balanced by any baseline risk factors due to the process of randomisation.

On the one hand, the results are encouraging as they suggest that culture results over the first few months of treatment can be an acceptable surrogate endpoint in certain trials in certain populations. However, it is unclear which populations this would apply to, as the heterogeneity in results could be due to a number of factors, not just the geographical location of trial sites.

More of the treatment comparisons in the Hong Kong trials were of two regimens that included rifampicin throughout and many regimens included intermittent dosing, even in the intensive phase of treatment. Culture conversion was also earlier on average in the Hong Kong trials as compared to the East African trials as has been noted elsewhere[3], this was probably due at least in part to the greater use of rifampicin in the Hong Kong trials. Delayed culture conversion has however been reported in African patients compared to non-African patients, albeit in liquid media, in a recent multi-site clinical trial[29]. The authors concluded the differences could be due to ‘modest variation in laboratory processes’ but that further investigation was needed to find other possible causes[29]. Studies have also shown cavitation to be strong risk factor for relapse[3], [14]; and differences have been seen in relapse rates[30] and culture conversion after eight weeks of treatment[31] by strain and lineage of *M. tuberculosis*, with a corresponding association between strain and ethnicity or geographical location.

Based on these results, a surrogate endpoint that is a summary measure of the longitudinal profile of culture results over time is likely to be more useful than a culture result at a single time point.

Two approaches for capturing the longitudinal profile of culture results that have been proposed are (i) using a parameter from repeated measures modelling of culture results over time or (ii) summarising the time to stable culture conversion in a survival analysis. These approaches have been described elsewhere[32]. Both have been used in phase II TB clinical trials that are completed[33], [34] and ongoing (the former in the TB Alliance study NC-002, clinicaltrials.gov identifier NCT01498419 and the latter in PanACEA MAMS-TB, Pan African Clinical Trials Registry identifier PACTR201205000383208). There is, however, no evidence as yet that either approach will yield markers that are acceptable surrogate endpoints.

Cultures were only performed monthly in the BMRC trials and therefore neither repeated measures modelling nor an analysis of time to stable culture conversion can be conducted with these data. Data from multiple treatment comparisons across large multi-centre trials will be necessary for a formal evaluation of a marker as a surrogate endpoint. Some data will become available in the next few years as a several large phase III clinical trials will be finishing and reporting results. Adaptive trial designs and innovative clinical development pathways are critical to compensate for the current lack of suitable surrogate endpoints[35].

In the only other formal evaluation of any marker as a surrogate endpoint for treatment response in TB[36], the authors evaluated two month culture conversion as a surrogate endpoint using data from published report of trials conducted by the BMRC. They selected 30 pairs of regimens showing that the slope of the meta-regression line ‘was statistically significant (p<0.00001)’. There is some spread around the fitted line, but the authors do not give a figure for the R^2^_trial_ to allow the reader to judge whether this analysis shows two month culture conversion to be an acceptable surrogate. The authors concluded that two month culture conversion ‘should be a surrogate endpoint for the registration of new drugs for the treatment of TB.’ They were, however, unable to evaluate culture status at any other time as a surrogate, as it was only common in trial reports to publish the two month culture conversion rates, and there was no evidence that they had looked at effect modification by geographical region.

## Limitations

Apart from the limitation of the cultures only being available monthly, the cultures were done on solid media which is being used increasingly less in clinical trials. The results may well be different when liquid media are used due to the increased diagnostic sensitivity[21].

These trials were conducted before methodology was available to distinguish true endogenous relapse from exogenous reinfection caused by a new strain of *M. tuberculosis*. It is therefore possible that a number of the cases recorded as relapses were in fact as a result of reinfection. However, this number is likely to be few for two reasons. Firstly reinfections occur more frequently in HIV co-infected patients[37], [38] and these trials were conducted before the HIV epidemic. Secondly, and more importantly, many of these trials had up to 5 years of follow-up finding very few recurrences in the final 2–3 years of follow-up. Results at 5 years were consistent with those after 30 months suggesting minimal impact of the inclusion of possible cases of exogenous reinfection[3].

The two-stage analysis methodology did have some drawbacks. The estimates of the α_ij_ were assumed in the second stage to be without error with the variance of the estimates only entering the model through the weights. The α_ij_ and β_ij_ are estimated separately in the first stage and the correlation between the two is therefore assumed to be zero, which is probably not the case since the estimates are from the same group of trial participants. This will result in estimates of R^2^_trial_ slightly above or below the true values, but the impact is likely to be minimal.

All comparisons were of two treatments with differences in the first few months of treatment. Many comparisons also involved changes in regimens after the putative surrogate endpoint had been measured. Of the six points in the lower right quadrant of Figure 2A, evaluating the 2 month culture in East African trials, two were of comparisons where rifampicin was added beyond two months. Trials have shown that rifampicin throughout treatment is critical[39], but this cannot be reflected in the two month culture result. Restricting the analysis to all comparisons of two regimens that contained rifampicin throughout treatment gives considerably better results for the two month culture (R^2^_trial_ = 0.67 compared to R^2^_trial_ = 0.36), but not as good as in the sub-group of Hong Kong trials only (R^2^_trial_ = 0.86).

Fundamentally, a marker that is measured before the end of treatment cannot capture the full effect of the treatment regimen and can never therefore be a perfect surrogate. This can be illustrated in a trial comparing a six month regimen with rifampicin throughout with an eight month regimen with rifampicin for only the first two months[39]. The two-month intensive phase was unchanged and therefore the proportion of culture positive patients at two months was similar (17% and 14% respectively) but the proportion with unfavourable outcomes at the end of follow-up was significantly different (5% and 10% respectively, p<0.01). The optimum time for measuring a marker that could be a surrogate at the end of treatment. Unlike the situation in HIV where CD4 count or viral load can always be measured, TB patients are almost without exception negative on culture at the end of treatment (unless they have failed treatment or have an uninformative isolated positive) and therefore it is likely that more sensitive methodologies that can detect the presence of *M. tuberculosis* later in treatment will be needed. These could include a molecular viable count assay[40], resuscitation-promoting factors[41] or cycle threshold of the Xpert MTB/RIF assay[42], [43].

## Conclusions

Without a better understanding of the main cause of the heterogeneity of results, neither the two month nor the three month culture on solid media can be recommended for use as the primary endpoint in a phase III clinical trial - the ultimate objective for a putative surrogate.

The results are encouraging, however, in that culture results on solid media during treatment capture a moderate proportion of the treatment effect on long-term outcome and are appropriate as endpoints for phase II trials to identify promising regimens to take forward to phase III for more rigorous evaluation. An endpoint that is a summary measure of the longitudinal profile of culture results over time or that is able to detect the presence of *M. tuberculosis* later in treatment is more likely to be a better endpoint for a phase II trial than a culture result at a single time point and may prove to be an acceptable surrogate. More data are needed before any endpoint can be used as a surrogate in a confirmatory phase III trial.

## References

[1] Youmans GP (1979) Tuberculosis. Philadelphia: W. B. Saunders.

[2] World Health Organization (2011) Global tuberculosis control 2011. Geneva.

[3] Fox W, Ellard GA, Mitchison DA (1999) Studies on the treatment of tuberculosis undertaken by the British Medical Research Council Tuberculosis Units, 1946–1986, with relevant subsequent publications. Int J Tuberc Lung Dis 3: S231–S279. 10529902

[4] Karim SSA, Churchyard GJ, Karim QA, Lawn SD (2009) HIV infection and tuberculosis in South Africa: an urgent need to escalate the public health response. Lancet 374: 921–933. 19709731 10.1016/S0140-6736(09)60916-8 PMC2803032

[5] Merle CS, Sismanidis C, Bah Sow O, Gninafon M, Horton J, et al (2012) A pivotal registration phase III, multicenter, randomized tuberculosis controlled trial: design issues and lessons learnt from the Gatifloxacin for TB (OFLOTUB) project. Trials 13: 61. 22607233 10.1186/1745-6215-13-61 PMC3528451

[6] Biomarker Definitions Working Group (2001) Biomarkers and surrogate endpoints: preferred definitions and conceptual framework. Clin Pharmacol Ther 69: 89–95. 11240971 10.1067/mcp.2001.113989

[7] Prentice RL (1989) Surrogate endpoints in clinical trials: definition and operational criteria. Stat Med 8: 431–440. 2727467 10.1002/sim.4780080407

[8] Nahid P, Saukkonen J, Mac Kenzie WR, Johnson JL, Phillips PP, et al (2011) CDC/NIH Workshop. Tuberculosis biomarker and surrogate endpoint research roadmap. Am J Respir Crit Care Med 184: 972–979. 21737585 10.1164/rccm.201105-0827WS PMC3208659

[9] Baker SG, Kramer BS (2003) A perfect correlate does not a surrogate make. BMC Med Res Methodol 3: : 16. Epub 2003 Sep 2009. 10.1186/1471-2288-3-16 PMC212489 12962545

[10] Perrin FMR, Lipman MCI, McHugh TD, Gillespie SH (2007) Biomarkers of treatment response in clinical trials of novel antituberculosis agents. Lancet Infect Dis 7: 481–490. 17524807 10.1016/S1473-3099(07)70112-3

[11] Nahid P, Saukkonen J, Mac Kenzie W, Johnson JL, Phillips PJ, et al.. (2011) Tuberculosis Biomarker and Surrogate Endpoint Research Roadmap. Am J Respir Crit Care Med. 10.1164/rccm.201105-0827WS PMC3208659 21737585

[12] Wallis RS (2007) Surrogate markers to assess new therapies for drug-resistant tuberculosis. Expert Rev Anti Infect Ther 5: 163–168. 17402829 10.1586/14787210.5.2.163

[13] Benator D, Bhattacharya M, Bozeman L, Burman W, Cantazaro A, et al (2002) Rifapentine and isoniazid once a week versus rifampicin and isoniazid twice a week for treatment of drug-susceptible pulmonary tuberculosis in HIV-negative patients: a randomised clinical trial. Lancet 360: 528–534. 12241657 10.1016/s0140-6736(02)09742-8

[14] Aber VR, Nunn AJ (1978) Short term chemotherapy of tuberculosis. Factors affecting relapse following short term chemotherapy. Bull Int Union Tuberc 53: 276–280. 387141

[15] Mitchison DA (1996) Modern methods for assessing the drugs used in the chemotherapy of mycobacterial disease. Soc Appl Bacteriol Symp Ser 25: 72S–80S. 8972122

[16] Mitchison DA (1993) Assessment of new sterilizing drugs for treating pulmonary tuberculosis by culture at 2 months. Am Rev Respir Dis 147: 1062–1063. 8466107 10.1164/ajrccm/147.4.1062

[17] Zumla A, Wallis R, Doherty M, Klein N, Parida S, et al (2008) Joint TDR/EC expert consultation on biomarkers in tuberculosis: Report of the joint TDR/EC expert consultation to evaluate the potential roles of biomarkers in the management of HIV-infected and HIV-uninfected patients with tuberculosis. Geneva

[18] Horne DJ, Royce SE, Gooze L, Narita M, Hopewell PC, et al (2010) Sputum monitoring during tuberculosis treatment for predicting outcome: systematic review and meta-analysis. Lancet Infect Dis 10: 387–394. 20510279 10.1016/S1473-3099(10)70071-2 PMC3046810

[19] Christie DA, Tansey EM (2005) Short-Course Chemotherapy for Tuberculosis: The transcript of a witness seminar held by the Wellcome Trust Centre for the History of Medicine at UCL, London, 3rd Febuary 2004.editor: Wellcome Trust Centre for the History of Medicine at UCL.

[20] D'Esopo ND (1982) Clinical trials in pulmonary tuberculosis. Am Rev Respir Dis 125: 85–93. 10.1164/arrd.1982.125.3P2.85 7041724

[21] Nunn AJ, Phillips PPJ, Gillespie SH (2008) Design issues in pivotal drug trials for drug sensitive tuberculosis (TB). Tuberculosis 88: S85–S92. 18762156 10.1016/S1472-9792(08)70039-8

[22] Phillips PP, Nunn AJ (2010) Challenges of Phase III study design for trials of new drug regimens for the treatment of TB. Future Medicinal Chemistry 2: 1273–1282 (1210).. 21426018 10.4155/fmc.10.221

[23] Daniels MJ, Hughes MD (1997) Meta-analysis for the evaluation of potential surrogate markers. Stat Med 16: 1965–1982. 9304767 10.1002/(sici)1097-0258(19970915)16:17<1965::aid-sim630>3.0.co;2-m

[24] Burzykowski T, Molenberghs G, Buyse ME (2005) The Evaluation of Surrogate Endpoints. New York: Springer.

[25] Global Alliance for TB Drug Development (2001) Tuberculosis. Scientific blueprint for tuberculosis drug development. Tuberculosis (Edinb) 81 Suppl 11–52. 11530398 10.1054/tube.2001.0288

[26] Sirgel FA, Donald PR, Odhiambo J, Githui W, Umapathy KC, et al (2000) A multicentre study of the early bactericidal activity of anti-tuberculosis drugs. J Antimicrob Chemother 45: 859–870. 10837441 10.1093/jac/45.6.859

[27] Spigelman MK (2007) New tuberculosis therapeutics: a growing pipeline. J Infect Dis 196 Suppl 1S28–S34. 17624823 10.1086/518663

[28] Wang JY, Wang JT, Tsai TH, Hsu CL, Yu CJ, et al (2010) Adding moxifloxacin is associated with a shorter time to culture conversion in pulmonary tuberculosis. Int J Tuberc Lung Dis 14: 65–71. 20003697

[29] Mac Kenzie WR, Heilig CM, Bozeman L, Johnson JL, Muzanye G, et al (2011) Geographic differences in time to culture conversion in liquid media: Tuberculosis Trials Consortium study 28. Culture conversion is delayed in Africa. PLoS One 6: e18358. 21494548 10.1371/journal.pone.0018358 PMC3073969

[30] Burman WJ, Bliven EE, Cowan L, Bozeman L, Nahid P, et al (2009) Relapse associated with active disease caused by Beijing strain of Mycobacterium tuberculosis. Emerg Infect Dis 15: 1061–1067. 19624921 10.3201/eid1507.081253 PMC2744226

[31] Nahid P, Bliven EE, Kim EY, Mac Kenzie WR, Stout JE, et al (2010) Influence of M. tuberculosis lineage variability within a clinical trial for pulmonary tuberculosis. PLoS One 5: e10753. 20505778 10.1371/journal.pone.0010753 PMC2873999

[32] Davies GR (2010) Early clinical development of anti-tuberculosis drugs: science, statistics and sterilizing activity. Tuberculosis (Edinb) 90: 171–176. 20382567 10.1016/j.tube.2010.03.007

[33] Rustomjee R, Lienhardt C, Kanyok T, Davies GR, Levin J, et al (2008) A Phase II study of the sterilising activities of ofloxacin, gatifloxacin and moxifloxacin in pulmonary tuberculosis. Int J Tuberc Lung Dis 12: 128–138 (111).. 18230244

[34] Dorman SE, Johnson JL, Goldberg S, Muzanye G, Padayatchi N, et al (2009) Substitution of Moxifloxacin for Isoniazid during Intensive Phase Treatment of Pulmonary Tuberculosis. Am J Respir Crit Care Med 180: 273–280. 19406981 10.1164/rccm.200901-0078OC

[35] Phillips PP, Gillespie SH, Boeree M, Heinrich N, Aarnoutse R, et al (2012) Innovative Trial Designs Are Practical Solutions for Improving the Treatment of Tuberculosis. J Infect Dis 10.1093/infdis/jis041 22448027

[36] Wallis RS, Wang C, Doherty TM, Onyebujoh P, Vahedi M, et al (2010) Biomarkers for tuberculosis disease activity, cure, and relapse. Lancet Infectious Diseases 10: 68–69. 20113972 10.1016/S1473-3099(10)70003-7

[37] Chaisson RE, Churchyard GJ (2010) Recurrent tuberculosis: relapse, reinfection, and HIV. J Infect Dis 201: 653–655. 20121432 10.1086/650531 PMC3407677

[38] Lambert M-L, Hasker E, Deun AV, Roberfroid D, Boelaert M, et al (2003) Recurrence in tuberculosis: relapse or reinfection? Lancet Infect Dis 3: 282–287. 12726976 10.1016/s1473-3099(03)00607-8

[39] Jindani A, Nunn AJ, Enarson DA (2004) Two 8-month regimens of chemotherapy for treatment of newly diagnosed pulmonary tuberculosis: international multicentre randomised trial. Lancet 364: 1244–1251. 15464185 10.1016/S0140-6736(04)17141-9

[40] Honeyborne I, McHugh TD, Phillips PP, Bannoo S, Bateson A, et al (2011) Molecular Bacterial Load Assay, a Culture-Free Biomarker for Rapid and Accurate Quantification of Sputum Mycobacterium tuberculosis Bacillary Load during Treatment. J Clin Microbiol 49: 3905–3911. 21900522 10.1128/JCM.00547-11 PMC3209113

[41] Mukamolova GV, Turapov O, Malkin J, Woltmann G, Barer MR (2010) Resuscitation-promoting factors reveal an occult population of tubercle Bacilli in Sputum. Am J Respir Crit Care Med 181: 174–180. 19875686 10.1164/rccm.200905-0661OC PMC2809243

[42] Blakemore R, Nabeta P, Davidow AL, Vadwai V, Tahirli R, et al (2011) A multisite assessment of the quantitative capabilities of the Xpert MTB/RIF assay. Am J Respir Crit Care Med 184: 1076–1084. 21836139 10.1164/rccm.201103-0536OC PMC3208646

[43] Rachow A, Zumla A, Heinrich N, Rojas-Ponce G, Mtafya B, et al (2011) Rapid and accurate detection of Mycobacterium tuberculosis in sputum samples by Cepheid Xpert MTB/RIF assay–a clinical validation study. PLoS One 6: e20458. 21738575 10.1371/journal.pone.0020458 PMC3126807

[44] East and Central African/British Medical Research Council (1983) Controlled clinical trial of 4 short-couse regimens of chemotherapy (three 6-month and one 8-month) for pulmonary tuberculosis. Tubercle 64: 153–166. 6356538 10.1016/0041-3879(83)90011-9

[45] Madras Tuberculosis Chemotherapy Centre (1960) A concurrent comparison of isoniazid plus PAS with three regimens of isoniazid alone in the domiciliary treatment of pulmonary tuberculosis in South India. Bull WHO 23: 535–585. 14447270 PMC2555609

[46] Simon G (1966) Radiology in epidemiological studies and some therapeutic trials. British Medical Journal 2: 491. 4161804 10.1136/bmj.2.5512.491 PMC1943538

